# Characterization of the CRISPR1-Cas array and its subtyping potential in Enterococcus faecalis from Malaysia

**DOI:** 10.1099/acmi.0.001070.v3

**Published:** 2026-01-30

**Authors:** Jia Qi Beh, Nazmul Hasan Muzahid, Jar Hui Mar, Calvin Bok Sun Goh, Marie Andrea Laetitia Huët, Shu Yong Lim, Sadequr Rahman

**Affiliations:** 1 School of Science, Monash University Malaysia, 47500, Bandar Sunway, Selangor Darul Ehsan, Malaysia; 2 Genomics Facility, Monash University Malaysia, 47500, Bandar Sunway, Selangor Darul Ehsan, Malaysia

**Keywords:** CRISPR-Cas, *Enterococcus faecalis*, epidemiology, MLST, repeat-spacer, typing

## Abstract

*Enterococcus faecalis* is a gram-positive bacterium and a common cause of hospital-associated infections. Three major CRISPR loci have been discovered in this species, namely, CRISPR1-*cas*, CRISPR2 and CRISPR3-*cas*. We developed novel primers which target the CRISPR1-*cas* loci in *E. faecalis* and tested these primers on 26 *E. faecalis* isolates isolated from diverse settings from Segamat, Malaysia. Half of the isolates were found to carry the CRISPR1-*cas9* locus, and the CRISPR1 array was successfully amplified in 12 out of 13 isolates that contained the *cas9* gene. Characterization of the CRISPR array shows that CRISPR1-*cas* shares similar array length and typical repeat sequences with CRISPR2 but differs significantly in terms of spacer identities and terminal repeat (TR) sequences. Most CRISPR spacers encode for chromosomal DNA sequences. Genotype characterization based on ancestral spacer (AS) and TR sequences indicates that *E. faecalis* with the same CRISPR1-AS genotype do not always harbour the same CRISPR2-AS genotypes and vice versa. A combined CRISPR1-*cas* and CRISPR2 typing offers comparable discriminatory power to MLST, suggesting its potential to be used in short-term strain identification and epidemiological surveillance at a lower sequencing cost. Our study provides a genetic reference for future studies in Southeast Asia.

## Data Availability

All data generated or analysed during this study are provided in supplementary materials with the online version of the article.

## Introduction

*Enterococcus faecalis* is a Gram-positive gut commensal bacterium known for its multidrug resistance (MDR) and for causing healthcare-associated infections [1,2]. Recent studies on *E. faecalis* have found a negative correlation between the occurrence of CRISPR (**C**lustered **R**egularly **S**paced **P**alindromic **R**epeats) loci and MDR, indicating the possible role of these loci in the evolution of this species [1]. Additionally, the potential of CRISPR sequences to be used in molecular typing has been suggested in several studies [1,3,5].

Most CRISPR-Cas loci are made up of two components – the repeat-spacer (CRISPR) array and an adjacent *cas* operon [6,7]. The presence of the *cas* operon is essential for the interference of CRISPR, and orphan CRISPRs were thought to lack the ability of incorporating new sequences from viral or mobile genetic elements (MGEs) [6]. On the other hand, in active CRISPR-Cas systems, the CRISPR array consists of alternating repeat and spacer subunits where the latter can be gained and lost with time [8]. Upstream of the repeat-spacer array, a conserved, AT-rich sequence known as the leader acts as the promoter for the transcription of the CRISPR array [9]. Repeat sequences range from 21 to 48 nt in different bacteria and are generally divided into two types – terminal repeats (TRs) and typical repeats. TR refers to the repeat sequence located closest to the leader-distal end of an array and directly adjacent to the ancestral spacer (AS) or the most ancient spacer, whereas typical repeat refers to all other non-TRs [10]. In contrast, spacer sequences can be 26 to 72 nt long in different bacteria and are more variable than repeat sequences [10,11]. Due to the variability in their spacers' sequence and number, the CRISPR arrays are known to serve as ‘molecular markers’ in some prokaryotic species and have been utilized as a typing or subtyping tool in a number of pathogenic bacteria [12,13].

In 2008, whole-genome analysis of *E. faecalis* strain OG1RF revealed the presence of two CRISPR loci [14]. Subsequent analysis of *E. faecalis* genomes showed that one of the loci, the CRISPR1-Cas, was associated with a type II-A *cas* operon (containing genes *cas1*, *cas2*, *cas9* and *csn2*), while the other CRISPR2 is an orphan locus lacking neighbouring *cas* genes [14]. Two years later, Palmer and Gilmore [1] conducted a thorough analysis of CRISPRs using available *E. faecalis* genomes and identified a third locus, named CRISPR3-Cas. All three CRISPR loci were observed to be present on fixed chromosomal locations across different *E. faecalis* strains. Since their initial discovery and analyses, CRISPR-Cas systems have been reported in only very few studies of *E. faecalis* [3,5,15, 16]. A number of observations were highlighted during the investigation of *E. faecalis* CRISPR loci in these reports. Firstly, all three CRISPR loci are located on fixed chromosomal locations, indicating that these loci might have originated from a common ancestor. Secondly, the orphan CRISPR2 array, which was hypothesized to be an inactive CRISPR due to the lack of an adjacent cas operon, is present in nearly all *E. faecalis* strains. Thirdly, the cas-associated CRISPR1 array shares highly similar repeat sequences with the orphan CRISPR2 array, suggesting their common ancestral origin. These raised the following question: Could these CRISPR loci, when used together, serve as an alternative method for local epidemiological typing, particularly in strain identification and subtyping? The CRISPR3-cas locus is relatively uncommon.

In this article, we investigate the diversity of CRISPR arrays in *E. faecalis* isolated from diverse but geographically co-located settings. We investigate the association of CRISPR1*-cas* and CRISPR2 arrays in *E. faecalis* and their association with MLST genotypes. This is the first study of CRISPR-Cas systems in *E. faecalis* isolated in Malaysia, emphasizing the characterization of repeat-spacer properties of the CRISPR1-Cas array in this species from this region. Characterization of orphan CRISPR2 arrays has been carried out extensively for this species due to primer availability, but the lack of primers for the CRISPR1-Cas arrays has been limiting studies for this locus. The overall aim of this study was to investigate the utility of using combined CRISPR1-Cas and CRISPR2 arrays for molecular typing of *E. faecalis* which could assist future epidemiological and evolutionary studies of this species.

## Methods

### Bacterial isolation and identification

This project was carried out as part of the Malaysian Microbiome Project, in collaboration with the South East Asia Community Observatory (SEACO) field team, Monash University Malaysia. A total of 26 *E. faecalis* isolates were obtained from the community faecal (*N*=19), environmental faecal (*N*=6) and clinical patient (*N*=1) samples. Community and environmental samples were collected from three sub-districts of Segamat (Bekok, Chaah and Jabi), whereas the single patient isolate was obtained from a patient with urinary tract infection from Hospital Segamat. Presumptive enterococci were selectively isolated on Bile Esculin Azide agar (Oxoid, UK) and stored at −80 °C in 25% glycerol. A routine culture was performed on Brain Heart Infusion Agar (Oxoid) and incubated overnight at 37 °C. *E. faecalis* isolates were phenotypically identified as Gram-positive, catalase-negative isolates and further confirmed by PCR using primers targeting the species-specific *sodA* genes in *E. faecalis* [17] with 16S rRNA as the DNA positive control. DNA extraction was performed on all isolates using Wizard Genomic DNA Purification Kit (Promega, USA).

### Antibiotic susceptibility profiling

The antibiotic susceptibility of *E. faecalis* isolates was assessed against ten antibiotics using the Kirby–Bauer disc diffusion method according to the Clinical and Laboratory Standard Institute (CLSI) guidelines [18]. The antibiotics used in the study were nitrofurantoin, F300; chloramphenicol, C30; ciprofloxacin, CIP5; erythromycin, E15; penicillin, P10; tetracycline, TE30; linezolid, LZD30; streptomycin, S300; gentamicin, CN120; and vancomycin, VA30. *Escherichia coli* ATCC BAA 2325 with known resistance patterns were used in the study as controls. For this profile, three technical and biological replicates were used.

### Identification of CRISPR arrays

Identification of two *cas-*associated loci, CRISPR1-Cas and CRISPR3-Cas, in *E. faecalis* isolates was performed via PCR amplification designed to bind to internal regions of *cas9* gene [1]. Fig. S1 (available in the online Supplementary Material) illustrates the structure of a typical type II CRISPR-Cas locus in *E. faecalis*. Isolates with negative PCR results were further tested for the absence of *cas9* using primers targeting the flanking regions of these loci due to the possibility of divergence of *cas9* sequences which could give misleading results. In *cas9*-negative isolates, the genomic location of CRISPR-Cas loci would be replaced by a short, conserved intergenic sequence (~300 bp) which could be identified as the PCR products following amplifications with primers EF0672-EF0673 for CRISPR1-null region and EF1760-EF1759 for CRISPR3-null region [1]. The cycling protocols for all CRISPR amplifications include an initial denaturation step at 94 °C for 5 min, followed by 30 cycles of denaturation at 94 °C for 1 min, annealing at respective temperatures for 1 min, extension at 72 °C for 1 min and a final extension step at 72 °C for 10 min. PCR products were analysed on 1.2% agarose gel in 1X Tris-borate-EDTA buffer and visualized using Gel Doc EZ Gel Imager (Bio-Rad, USA).

Following the identification of the CRISPR1-*cas9* gene, PCR was performed to amplify the CRISPR1 arrays of all *cas9*-positive isolates and orphan CRISPR2 array in all isolates. CRISPR3-Cas array could not be analysed due to its absence in all isolates tested. Amplification of the CRISPR1 array was performed using novel primers targeting the upstream and downstream regions of the array and was designed based on genome sequence alignment of *E. faecalis* strains with (OG1RF, GenBank accession: GCF_000172575.2) and without (D32 and L8, GenBank accessions: GCF_000281195.1 and GCF_009498175.3) the array. CRISPR1-Cas sequences were downloaded from the NCBI RefSeq database. On the other hand, PCR amplification for the CRISPR2 array was performed using primers targeting the flanking regions EF2063 and EF2061 [1]. For both amplifications, PCR cycling protocols for CRISPR arrays were the same as for the *cas9* genes, with an exception for the annealing temperatures (Supplementary Material 1, Table S1). PCR products obtained were run on 1.5% agarose gels and visualized.

### Characterization of CRISPR repeat-spacer sequences

Following successful amplification, PCR products from both CRISPR1 and CRISPR2 array amplifications were used for Sanger sequencing. The resulting sequencing products in FASTA format were used as inputs for CRISPRCasFinder (https://crisprcas.i2bc.paris-saclay.fr/CrisprCasFinder/Index) [19] to identify the repeat and spacer sequences. The spacer identities were then compared against the GenBank sequences using Nucleotide blast (blastn) of the NCBI nonredundant nucleotide database with default parameters for short input sequences. Given the short spacer sequences (<35 bp), only hits with ≥90% identity (27 of 30 nucleotides) were considered significant.

### Statistical analysis of association

The association between CRISPR-Cas and antibiotic resistance has been previously established in several studies [1,20]. In this study, the relationship between CRISPR-Cas and antibiotic resistance phenotypes was examined using chi-squared test available on IBM SPSS Statistics v26 [21], to determine whether CRISPR-Cas-positive isolates are associated with MDR or resistance to individual antibiotic. A *P*-value of <0.05 indicates statistical significance.

### Molecular typing by CRISPR array

CRISPR typing was performed using CRISPR1 and CRISPR2 arrays following sequencing. Repeat sequences were omitted from the primary CRISPR array sequences to generate a length of continuous spacer sequences. Each unique spacer was assigned an identifier number, and the entire length of spacers was concatenated to obtain a unique CRISPR spacer dictionary for each individual isolate (i.e. **1**-2-3-5-7). Isolates were then grouped into different AS genotypes (highlighted in bold) according to their AS sequences (spacer sequence adjacent to the TR). Isolates which share more spacers within the same AS genotype will be considered as more phylogenetically related, in other words, sharing more recent ancestors. In addition, the TR sequences from each primary CRISPR array sequence were also compared to determine whether they are concordant with the other isolates with the same AS genotype. For comparisons within and between CRISPR1 and CRISPR2 loci, only the TRs but not the typical repeats (or non-TRs) were used as the criteria for genotypic designation as typical repeats can exist in more than one variant in a single CRISPR array.

### Molecular typing by MLST

Out of the 26 isolates obtained, a total of 17 isolates were chosen for MLST, which include primarily isolates with both CRISPR1 and CRISPR2 loci (*N*=12), and additional isolates that either (i) have identical CRISPR2 loci (*N*=4) or (ii) were recovered from the same individual(s) or sample(s) (*N*=4). MLST for *E. faecalis* was performed by PCR amplification of seven target housekeeping genes, *aroE*, *gdh*, *gki*, *gyd*, *pstS*, *xpt* and *yqiL* using target-specific primers [22]. After obtaining the allele sequences, each allele was aligned with the reference alleles downloaded from the PubMLST database for *E. faecalis* (https://pubmlst.org/organisms/enterococcus-faecalis) [22] and trimmed to obtain the consensus sequences, which are then concatenated and submitted to the *E. faecalis* MLST database. A sequence type (ST) was assigned based on the unique combination of allele types. Isolates with identical allele combinations have the same ST, whereas isolates with one or two allele differences are known as single- and double-locus variants, respectively.

### Comparative analysis of typing methods

The following comparative analyses were carried out using isolates from Segamat and complete *E. faecalis* genomes obtained from RefSeq database. Complete *E. faecalis* genomes were downloaded from RefSeq genome database for *E. faecalis* (ID 808) (https://www.ncbi.nlm.nih.gov/datasets/genome/?taxon=1351) and their CRISPR arrays and STs were identified. A complete list of *E. faecalis* genomes used and their CRISPR genotypes is available in Supplementary Material 2. All statistical tests in the following sections were performed using an open-source, online tool at comparing partitions (http://www.comparingpartitions.info/index.php?link=%20Tool). A 95% CI was used as an indicator of significant differences between typing methods using SPSS Statistics [21].

Typical and TR sequences from CRISPR1 and CRISPR2 arrays, obtained from both Segamat and RefSeq genomes, were aligned using mega-X [23] to generate consensus sequences for each locus. Statistical tests were performed to examine the correspondence of CRISPR1 genotypes to CRISPR2 genotypes. The correspondence between both arrays was assessed by looking at the concordance between their AS and TR sequences. This correspondence was assessed using the adjusted Rand (AR) index, an indicator used to assess global congruence between genotypes [24]. Values of AR range from 0 to 1, with higher values indicating greater concordance.

Comparative analysis of CRISPR and MLST genotypes was performed using all isolates with pre-determined CRISPR2 genotypes and STs. Multiple sequence alignment and phylogenetic tree construction were carried out using Molecular Evolutionary Genetic Analysis (mega 12) software (v12) [25]. Maximum-likelihood phylogenetic trees were constructed from the concatenated MLST and CRISPR sequences in mega, applying the GTR+G (general time reversible+Gamma) substitution model to provide a visual representation of all STs and CRISPR genotypes for the isolates analysed. Branch support was evaluated using 1,000 bootstrap replicates. Tree visualization and annotations were performed in the Interactive Tree of Life (iTOL) v6 [26]. Three CRISPR typing methods (CRISPR1, CRISPR2 and combined CRISPR1 and CRISPR2) were compared in terms of their discriminatory power and congruence with MLST genotypes. The discriminatory power of a typing method was assessed using the Simpson’s index of diversity (SID), with values ranging from 0 to 1 [27]. A higher SID value indicates greater diversity and potential to be used in species subtyping. Adjusted Wallace (AW) coefficient was used to determine how well CRISPR genotypes predict their corresponding STs in MLST [28]. In contrast to the AR indices, which indicate a unidirectional relationship between two genotypes, the AW provides an asymmetrical (two-way) interpretation of congruence between typing methods, i.e. by looking at AWMLST→CRISPR and AWCRISPR→MLST.

## Results

### MDR *E. faecalis* identified from community and environmental sources

The analysis of antibiotic resistance revealed that five isolates were MDR: three isolates from the community and two from the environment (Table 1). The only hospital isolate was not resistant against any of the antibiotics tested. Out of 26 isolates, 9 were resistant against tetracycline. In addition, 6/26 and 5/26 were resistant against ciprofloxacin and chloramphenicol, respectively. This data suggests that the community and the environment in Malaysia are reservoirs for MDR *E. faecalis* isolates.

**Table 1.** Antibiotic resistance profile of 26 *E. faecalis* isolates from community, environmental and clinical samples

Isolate		Zone of inhibition, in diameter (mm)										
		F300	C30	CIP5	E15	P10	TE30	LZD30	S300	CN120	VA30	Resistant to antibiotics
Community isolates	C12	22 (S)	6 (R)	6 (R)	15 (I)	15 (S)	25 (S)	14 (R)	6 (R)	6 (R)	16 (I)	5 (MDR)
	C29	25 (S)	22 (S)	22 (S)	17 (I)	16 (S)	30 (S)	25 (S)	21 (S)	20 (S)	17 (S)	
	C34	25 (S)	24 (S)	21 (S)	20 (I)	18 (S)	30 (S)	21 (I)	18 (S)	20 (S)	18 (S)	
	C59	25 (S)	24 (S)	22 (S)	20 (I)	22 (S)	27 (S)	6 (R)	22 (S)	22 (S)	18 (S)	1
	C63-1	26 (S)	24 (S)	22 (S)	20 (I)	19 (S)	29 (S)	25 (S)	21 (S)	21 (S)	17 (S)	
	C63-2	20 (S)	20 (S)	19 (I)	18 (I)	21 (S)	22 (S)	24 (S)	16 (S)	19 (S)	17 (S)	
	C74	25 (S)	24 (S)	22 (S)	20 (I)	21 (S)	30 (S)	24 (S)	23 (S)	23 (S)	18 (S)	
	C90	23 (S)	21 (S)	18 (I)	25 (S)	21 (S)	24 (S)	24 (S)	15 (S)	17 (S)	17 (S)	
	C104	22 (S)	20 (S)	18 (I)	22 (I)	21 (S)	21 (S)	23 (S)	15 (S)	18 (S)	17 (S)	
	C107	22 (S)	19 (S)	18 (I)	23 (S)	21 (S)	22 (S)	24 (S)	16 (S)	20 (S)	17 (S)	
	C108	26 (S)	11 (R)	25 (S)	6 (R)	19 (S)	8 (R)	25 (S)	20 (S)	22 (S)	18 (S)	3 (MDR)
	C113	27 (S)	14 (I)	25 (S)	6 (R)	18 (S)	9 (R)	25 (S)	21 (S)	21 (S)	18 (S)	2
	C119	25 (S)	23 (S)	20 (I)	20 (I)	18 (S)	29 (S)	28 (S)	21 (S)	23 (S)	19 (S)	
	C126	26 (S)	21 (S)	21 (S)	20 (I)	20 (S)	30 (S)	27 (S)	23 (S)	23 (S)	18 (S)	
	C127	25 (S)	11 (R)	20 (I)	6 (R)	18 (S)	8 (R)	26 (S)	22 (S)	22 (S)	18 (S)	3 (MDR)
	C157	21 (S)	23 (S)	16 (I)	16 (I)	18 (S)	11 (R)	6 (R)	21 (S)	21 (S)	16 (I)	2
	C195	27 (S)	25 (S)	24 (S)	19 (I)	16 (S)	33 (S)	30 (S)	23 (S)	25 (S)	18 (S)	
	C196	24 (S)	24 (S)	21 (S)	21 (I)	16 (S)	20 (S)	27 (S)	24 (S)	23 (S)	17 (S)	
	C201	26 (S)	24 (S)	21 (S)	20 (I)	16 (S)	30 (S)	29 (S)	23 (S)	24 (S)	18 (S)	
Environmental isolates	EBC10-1	23 (S)	18 (S)	19 (I)	6 (R)	22 (S)	6 (R)	25 (S)	18 (S)	21 (S)	16 (I)	2
	EBC10-3	18 (S)	21 (S)	21 (S)	19 (I)	20 (S)	6 (R)	25 (S)	22 (S)	25 (S)	17 (S)	1
	EBFW30	22 (S)	11 (R)	10 (R)	6 (R)	19 (S)	8 (R)	25 (S)	6 (R)	20 (S)	17 (S)	5 (MDR)
	EBM6	23 (S)	22 (S)	21 (S)	26 (S)	18 (S)	6 (R)	27 (S)	20 (S)	22 (S)	18 (S)	1
	ECC16	25 (S)	8 (R)	22 (S)	6 (R)	16 (S)	9 (R)	24 (S)	19 (S)	6 (R)	18 (S)	4 (MDR)
	ECR1	25 (S)	25 (S)	24 (S)	25 (S)	14 (R)	30 (S)	27 (S)	21 (S)	21 (S)	16 (I)	1
Clinical isolates	H11221	22 (S)	20 (S)	19 (I)	23 (S)	22 (S)	23 (S)	24 (S)	15 (S)	18 (S)	16 (I)	

C30, chloramphenicol; CIP5, ciprofloxacin; CN120, gentamicin; E15, erythromycin; F300, nitrofurantoin; I, intermediate; LZD30, linezolid; P10, penicillin; R, resistant; S300, streptomycin; S, susceptible; TE30, tetracycline; VA30, vancomycin.

### The CRISPR1 locus was detected in half of the Segamat isolates

A total of 26 *E. faecalis* isolates from community (*N=*19), environmental (*N*=6) and clinical (*N=1*) samples were employed in this study. None of the isolates were PCR-positive for the CRISPR3-*cas9* gene. Following the identification of *cas9* genes, the CRISPR arrays of CRISPR1-Cas and orphan CRISPR2 loci were amplified using specific primers to study their repeat-spacer properties. The CRISPR1 array was successfully amplified in 12 out of 13 isolates tested positive for CRISPR1-*cas9* gene, whereas the CRISPR2 array was detected in all 26 isolates. Using PCR, we were able to identify a single variant of CRISPR1 and/or CRISPR2 array in each isolate. The length of the *cas*-associated CRISPR1 array typically ranged from 0.1 to 1.1 kb, whereas orphan CRISPR2 arrays were 0.1–0.9 kb long. The average number of spacers for CRISPR1 and CRISPR2 arrays was 6.3 (range: 1 to 15) and 4.8 (range: 1 to 12), respectively. The majority of spacer and repeat sequences of both arrays have identical lengths of 30 and 36 nt, respectively, a signature length observed in type II-A CRISPR-Cas systems in bacteria [3]. No significant association was found between the presence (or absence) of CRISPR-Cas and MDR or resistance to individual antibiotic, except for erythromycin (Supplementary Material 1, Table S2).

The identities of spacers for each locus are summarized in Table 2. The majority of CRISPR2 spacers (72%) were found to be chromosomally encoded sequences, whereas most spacers in CRISPR1 were ‘unidentified regions’. Spacers containing sequences derived from the same MGE occurred in strains isolated from different samples, such as the sequences traced to enterococcal phage phiEF11 and phage vB. Some spacer sequences were apparently derived from shared structural genes between different viral families. Spacer sequences from the same viral families were identified in both CRISPR1 and CRISPR2 arrays of community isolate 119-1 and environmental isolate BM6-2, indicating that both strains might have been previously exposed to the same viral infection.

**Table 2.** Spacer identities of CRISPR1 and CRISPR2 arrays, based on the NCBI non-redundant nucleotide database

CRISPR array	Proportion of matching sequences against database (%)		
	Chromosomal	MGE	Unidentified
CRISPR1	13.8	13.8	72.3
CRISPR2	65.3	12.1	22.6

Spacers were grouped into sequences derived from chromosomal, MGE or unidentified sources.

### CRISPR typing offers equivalent discrimination to MLST for strain typing but involves less sequencing

Concordance between repeat sequences of the CRISPR1 and CRISPR2 arrays was studied using Segamat isolates and RefSeq genomes. Out of 50 RefSeq genomes (randomly selected), 36 isolates possess the orphan CRISPR2 locus and 14 isolates possess the CRISPR1-Cas locus (Supplementary Material 2). Based on the alignment of typical repeat sequences, similar consensus sequences can be obtained for CRISPR1 (GTTTTAGAGXCATGTTGTXTAXAATGXXACCAXAXC) and CRISPR2 (XTTTTAGAGXXATGTTGTTXXGAXTGXXXCXAAAAC), where X represents nucleotide substitution sites. As for the TRs, different consensus sequences were obtained for CRISPR1 (GTTTTAGAXXCATGTTGTTTAGTTTTCGCAXATACG) and CRISPR2 (GTTTTAXAGXCATGTTGTTXAAAAAXAAACTATCAC). These suggest that the TRs could be more representative of a particular CRISPR locus than the typical repeats in this species.

To assess the feasibility of using CRISPR arrays as a typing or subtyping tool, the spacer sequences were used to construct spacer dictionaries and AS genotypes for each locus (Table 3). A total of nine and seven different AS genotypes were obtained for CRISPR2 and CRISPR1 loci, respectively, among the Segamat isolates. Both CRISPR loci demonstrate diversity in spacers and TR sequences. Similarly, isolates with the same CRISPR1-AS genotype might have different CRISPR2-AS genotypes. Next, we assessed the concordance between CRISPR1 and CRISPR2 genotypes based on their AS and TR sequences. For comparative purposes, 25 isolates (12 Segamat and 13 RefSeq genomes) with both CRISPR loci were included in the analysis. Based on the AR indices, the highest congruence (0.547) was achieved between CRISPR2-AS and CRISPR2-TR. Other methods have coefficients less than 0.5. Congruence within CRISPR2 genotypes (CRISPR2 AS↔TR=0.547) is greater than within CRISPR1 genotypes (CRISPR1 AS↔TR=0.140). Congruence between TRs (0.218) is generally higher than between ASs (0.186), indicating that CRISPR1 and CRISPR2 loci are each more concordant in terms of their repeats rather than spacer genotypes.

**Table 3.** CRISPR1, CRISPR2 and MLST genotypes of the Segamat isolates

Isolate	CRISPR2 AS genotype	CRISPR2 TR genotype	CRISPR2 dictionary	CRISPR1 AS genotype	CRISPR1 TR genotype	CRISPR1 dictionary	MLST
**C12**	1	4	**1**-37-17-18-19-20-21	1	4	**1**-8-9-10-11	192
**C34**	1	4	**1**-37-25-26-27	1	4	**1**-9-10-11	466
**C157**	1	4	**1**-37-52-53-25-54-55s	n/a			n/a
**C196**	1	4	**1**-37-52-53-26-58	n/a			n/a
**CR1**	1	4	**1**-52-53-83	5	3	**5**-26-27-28-29-30-31	482
**C29**	2	3	**2**-22-23-24	n/a			n/a
**C63-1**	2	5	**2**-31-32-33-34-35	n/a			n/a
**C119**	2	5	**2**-42-43	Singleton	3	**3**-38-39-40-41	1,078
**BFW30**	2	5	**2**	n/a			n/a
**CC16**	2	4	**2**	n/a			n/a
**BC10-3**	2	1	**2**-71-72-73-74	5	3	**5**-32-33-34-35-36-37	373
**C90**	5	2	**5**-38	2	5	**2**-12-13-14-15-16	282
**C104**	5	2	**5**-38	2	5	**2**-12-13-14-15-16	282
**C107**	5	2	**5**-38	2	5	**2**-12-13-14-15-16	282
**BM6**	5	1	**5**-75-76-77-78-79-80-81-82	Singleton	3	**7**-45-46-47-48-49-50-51-52-53-54-55-56-57-58	674
**H11221**	5	2	**5**-75-76-76-77-84-85-86-87-88-89-90	2	1	**2**-17-18-19-20-21-22-23-24-25	387
**C108**	6	5	**6**-39-40-41	n/a			535
**C113**	6	5	**6**-39-40-41	n/a			535
**C127**	6	5	**6**-39-40-41	n/a			535
**C195**	6	4	**6**-56-57	n/a			n/a
**BC10-1**	6	5	**6**-56-67-68-69-40-70	Singleton	4	**6**-42-43-44	97
**C59**	Singleton	5	**3**-28-29-30-30	n/a			n/a
**C74**	Singleton	4	**4**	n/a			n/a
**C126**	Singleton	5	**7**-44-45-46-47-48-49-50-51	n/a			990
**C201**	Singleton	5	**8**-59-60-61-62-63-64-65-66	Singleton	2	**4**	1,079
**C63-2**	Singleton	5	**16**-36	n/a			852

N/A, not available.

Isolates were grouped according to their CRISPR2 AS genotypes, given that AS represents the most ancient spacer (or spacer adjacent to the TR). Twelve out of 13 CRISPR1 arrays were successfully amplified in the CRISPR1-*cas9*-positive isolates. MLST was conducted on selected isolates (*N*=17) due to funding limitations. Singletons represent isolates that do not share the same CRISPR AS genotype with any other isolates.

**Fig. 1.**
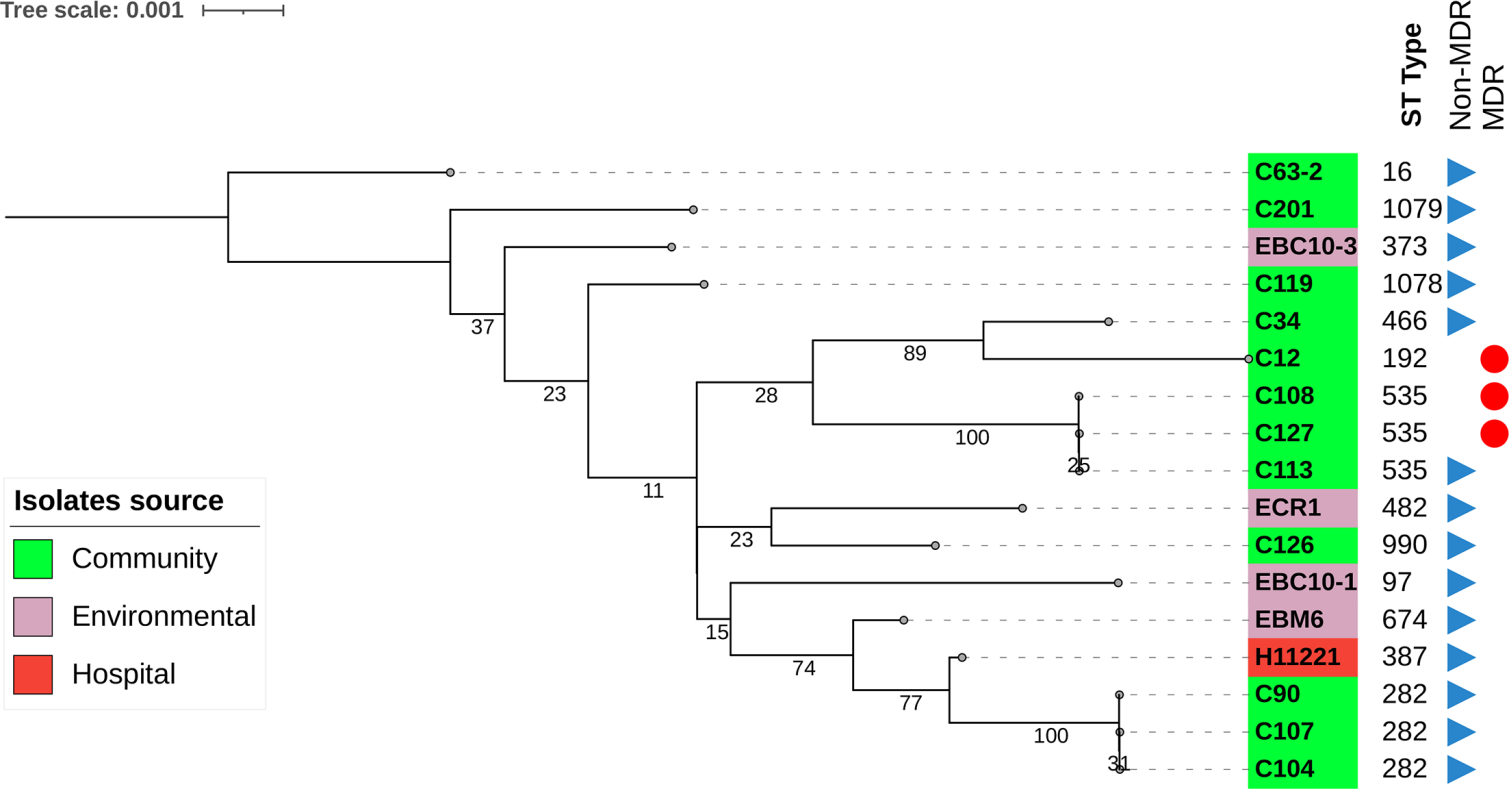
Maximum-likelihood tree generated based on concatenated MLST alleles. The source of the isolate was indicated in different colours – community (green), environmental (purple) and hospital (red). The tree was generated using mega 12 with default parameters applying the GTR+G (general time reversible+gamma) substitution model using 1,000 bootstrap replicates.

Following CRISPR characterization, MLST typing on 17 selected *E. faecalis* from Segamat revealed 13 different STs. Based on the maximum-likelihood tree, community, environmental and hospital isolates from Segamat were not clustered into distinct branches, suggesting that no clear partition exists between *E. faecalis* isolated from different sources (Fig. 1). This suggests that the isolates obtained from different locations across Segamat were distantly related to each other in terms of the number of shared MLST alleles.

**Fig. 2.**
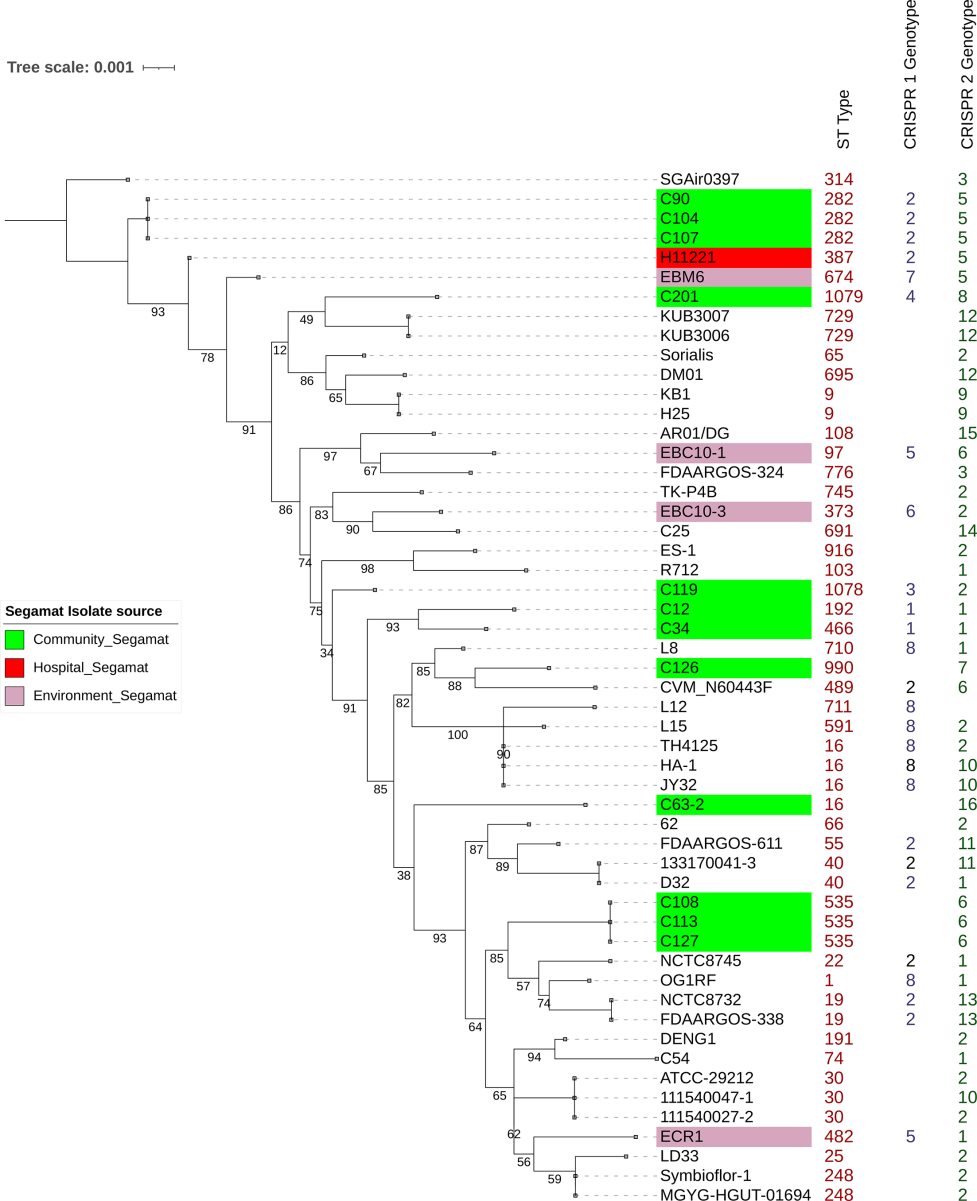
Maximum-likelihood tree generated for *E. faecalis* based on MLST sequences of Segamat (*N*=17) and RefSeq genomes (*N*=36). STs, CRISPR1 and CRISPR2 genotypes of the isolates were included on the right. Segamat isolates are indicated in green, red and purple for community, hospital and environmental isolates, respectively. The tree was generated using mega 12 with default parameters applying the GTR+G (general time reversible+gamma) substitution model using 1,000 bootstrap replicates.

A maximum-likelihood tree was then constructed using Segamat (*N*=17) and RefSeq (*N*=36) isolates based on concatenated MLST alleles (Fig. 2). According to the SID values, combined CRISPR typing (0.992) was shown to have the highest but similar discriminatory ability to CRISPR2 typing alone (0.991), and both have SIDs greater than MLST (0.985). This slight difference is probably due to the differences in sample size. Overlapping 95% CIs between MLST, CRISPR2 and combined CRISPR typing suggests that these methods do not differ significantly in terms of their discriminatory power. CRISPR1 reported the lowest SID (0.740) due to its absence in half of the isolates analysed. Based on the AW coefficients, the probability of two isolates having the same CRISPR2 genotype having identical MLST genotype is 81.5% (AWCRISPR2→MLST=0.815). Combined CRISPR1 and CRISPR2 typing has a lower predictive ability of 79.7%, followed by CRISPR1 (again, due to its absence in half of the isolates). Overall, the SID and AW values suggest that combined CRISPR typing shows a high discriminatory power equivalent to CRISPR2 alone, and both methods can be used to predict an isolate’s ST with over 80% congruency based on the small sample sizes in this study.

## Discussion

Twenty-six *E. faecalis* isolates from Segamat, Malaysia, were characterized for their CRISPR1 and CRISPR2 loci. All isolates in this study possess the orphan CRISPR2 locus, supporting the previous hypothesis that CRISPR2 is common in *E. faecalis*. In contrast, half of the isolates tested positive for CRISPR1-*cas9* and none were positive for CRISPR3-*cas9*. All isolates in this study possess the orphan CRISPR2 locus, supporting the previous hypothesis that CRISPR2 is common in *E. faecalis*. One of the community isolates, C63-1, was positive for the CRISPR1-*cas9* locus but failed to amplify using primers targeting CRISPR1 array despite repeated attempts, which could be due to mutations on the primer target site. Whole-genome sequencing and *in vitro* experiments would be useful to resolve this and to confirm the absence of the CRISPR3 loci in our study isolates and to confirm the validity of the CRISPR1 primers. Our study was also limited by the small sample size (only 26 *E. faecalis* isolates tested) and restricted geographical coverage and thus may not be representative at a broader population or species level. Investigations involving * E. faecalis* CRISPRs have been rare since their initial discovery in 2010. Lyons *et al*. [16] studied the prevalence of CRISPR1-Cas gene in commensal and environmental *Enterococcus* spp. and found that 23.6% of *E. faecalis* isolates possess the *cas* gene. The prevalence of CRISPR1- and CRISPR3-Cas among *E. faecalis* isolated from healthy pigs in Canada was reported to be similar (52% and 0%, respectively) to that observed in this study, indicating that CRISPR3 was generally rare in this species [15]. In contrast, a study in Brazil investigating CRISPRs in humans and animals showed that CRISPR1 and CRISPR3 were present in a surprisingly high percentage (46.7% and 67.4%, respectively) of all *Enterococcus* isolates, indicating that the prevalence of both CRISPR loci could vary in different countries [3]. The differences observed in CRISPR-Cas distribution could be attributed to the selective pressure of the local environment, from which antibiotic resistance and phage infections might have played a huge role in shaping CRISPR abundances and diversities.

Analysis of CRISPR2 arrays showed that the majority of spacers (>60%) were derived from the *E. faecalis* chromosome. Self-targeting spacers made up ~22% of spacers analysed in lactic acid bacteria, but the exact role of these spacers remains unknown [29]. Studies on *Streptococcus agalactiae* type II-A CRISPRs showed that 33% of spacers match the chromosome sequence [12]. Large-scale analysis of CRISPR arrays of more than 50,000 genomes revealed that ~6% of spacers have matches against prokaryotic genomes [30]. Earlier reports described self-targeting spacers as having a role in gene regulation by acting as small RNAs which prevent transcription of the complementary target gene [31]. Evidence of gene regulation has been reflected in the pathogenic bacterium *Pseudomonas aeruginosa*, in which the presence of spacer sequences identical to specific mRNAs ultimately leads to mRNA cleavage and immune evasion [32]. Others suggest that these spacers may play a role in species evolution, where self-targeting induces deletions of large genome segments, prompting extensive genome repair which subsequently leads to species divergence [11]. In contrast, the majority of spacers in CRISPR1 do not have close matches in the available nucleotide databases. The exact identities and functions of these CRISPR ‘unidentified regions’ remain unknown but could possibly represent non-coding regions or remnants of lysogenized phage in the bacterial genome. CRISPR1 and CRISPR2 loci exhibit similarities and differences in their CRISPR arrays. Spacer and repeat lengths are identical in both CRISPRs. Meanwhile, AS sequences from both loci demonstrated distinct identities. Where CRISPR2 arrays most often have chromosomal-encoding sequences as their ASs, this is rare for CRISPR1. Given its very common occurrence in *E. faecalis*, CRISPR2 was speculated to be acquired by this species at an earlier stage than CRISPR1.

In this study, we found that *E. faecalis* isolated from the same host could be of different strain type (STs) and harbour different CRISPR1 and/or CRISPR2 genotypes. Apart from showing that one strain co-inhabits with another strain, it also suggests that lateral exchange of CRISPR loci does not usually occur in the human or animal gut. However, it does not rule out the possibility of horizontal transfer of CRISPR loci given that these strains could have colonized the gut for transient periods. Analysis of the CRISPR1 and CRISPR2 spacer sequences showed that the loci do not share any identical spacer sequences, albeit they coexist in the same cell, implying independent spacer acquisition and the lack of spacer exchange between CRISPR loci. Notably, both loci were found to have overlapping typical repeat sequences, but the TRs were always locus-specific.

Comparison between strain typing methods suggests that combined CRISPR typing offers equivalent discrimination to MLST and was generally in congruence with the isolates’ STs, suggesting its possibility to be harnessed in strain identification or tracking in a localized setting. CRISPR typing may also provide a general glimpse of the prevalent phage or ‘MGE pool’ in a local environment. However, one drawback to CRISPR typing is the limited ability to align CRISPR arrays in order to construct an evolutionary rate based on nucleotide substitutions, unlike the housekeeping genes in MLST. Thus, CRISPR typing could be more useful in short-term epidemiological investigations, given that shorter regions need to be sequenced, whereas MLST is more suited for long-term evolutionary and phylogenetic studies.

## Conclusion

Twenty-six *E. faecalis* isolates from Segamat, Malaysia, were assessed for their CRISPR diversity and subtyping potential in this study. Results obtained supported the conclusions from previous studies that the orphan CRISPR2 locus occurred frequently in *E. faecalis*. CRISPR1 and CRISPR2 arrays were found to share similar repeat and spacer properties but are distinct in terms of their TR sequences, while their spacer identities suggest that isolates in Segamat possess distinct spacers to global strains at the CRISPR1 locus. A combination of CRISPR1 and CRISPR2 typing has been shown to possess comparative discrimination and concordance with MLST, supporting its potential to be harnessed as a subtyping tool at a lower sequencing cost in public health surveillance. Future studies should investigate the longitudinal properties of these CRISPR loci and their intra-species transferability, in order to provide a comprehensive understanding of species evolution in the community and healthcare settings.

## Supplementary material

10.1099/acmi.0.001070.v3 Uncited Supplementary Material 1.

10.1099/acmi.0.001070.v3 Uncited Supplementary Material 2.

## Abbreviations

AR: adjusted Rand
AS: ancestral spacer
AW: adjusted Wallace
MDR: multidrug resistance
MGE: mobile genetic element
SID: Simpson’s index of diversity
ST: sequence type
TR: terminal repeat
