# Supplementary material for: Characterization of the CRISPR1-Cas array and its subtyping potential in Enterococcus faecalis from Malaysia
Source: Access Microbiol. 2026 Jan 30;8(1):001070.v3. doi: 10.1099/acmi.0.001070.v3 (PMC12859217; doi:10.1099/acmi.0.001070.v3)
Supplement: Uncited Supplementary Material 1. [file acmi-8-01070-s001.pdf]

Supplementary Material - 1

Characterization of the CRISPR1-Cas array and its subtyping potential in *Enterococcus faecalis* from Malaysia

Jia Qi Beh[1], Nazmul Hasan Muzahid[1], Jar Hui Mar[1], Calvin Bok Sun Goh[1], Marie Andrea Laetitia Huët[1], Shu Yong Lim[2], Sadequr Rahman[1,2*]

[1]School of Science, Monash University Malaysia, 47500, Bandar Sunway, Selangor Darul Ehsan, Malaysia

[2]Genomics Facility, Monash University Malaysia, 47500, Bandar Sunway, Selangor Darul Ehsan, Malaysia

Corresponding author:

Nazmul Hasan Muzahid; Email: nazmul.muzahid26@gmail.com

Sadequr Rahman; Email: sadequr.rahman@monash.edu

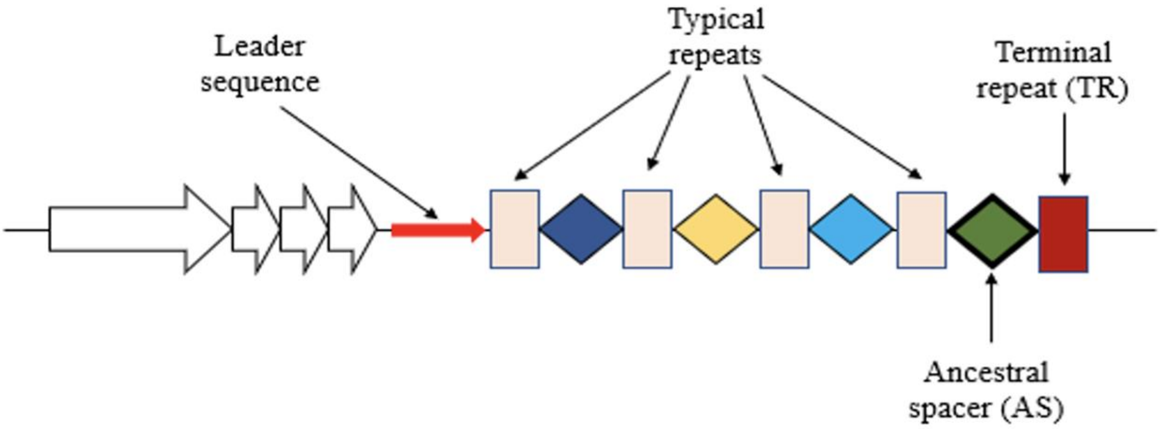

Figure S1: Diagram depicting the structure of a typical Type II CRISPR-Cas array (1). Ancestral Spacer (AS) represents the most ancient spacer acquired at an early stage, whereas new spacers are added at the leader-proximal end of the array. Leader sequence acts as the promoter where transcription is initiated. Coloured rectangles and diamonds represent repeat and spacer, respectively. White arrows indicate *cas* genes.

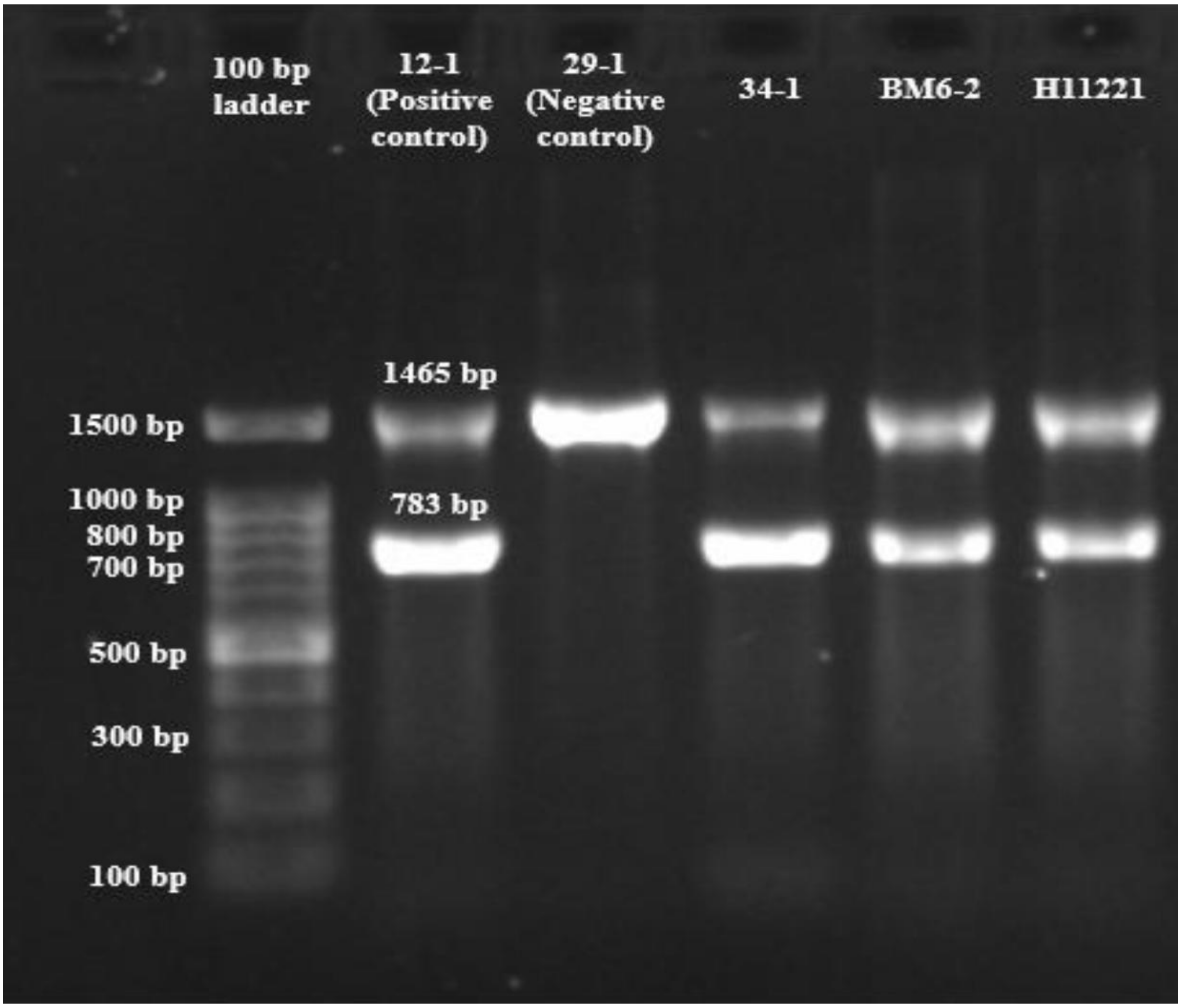

Figure S2: PCR amplification of internal regions of CRISPR1-*cas9* (783 bp). 16S rRNA control (1465 bp) was included. Isolates 12-1 and 29-1 were used as positive and negative controls, respectively.

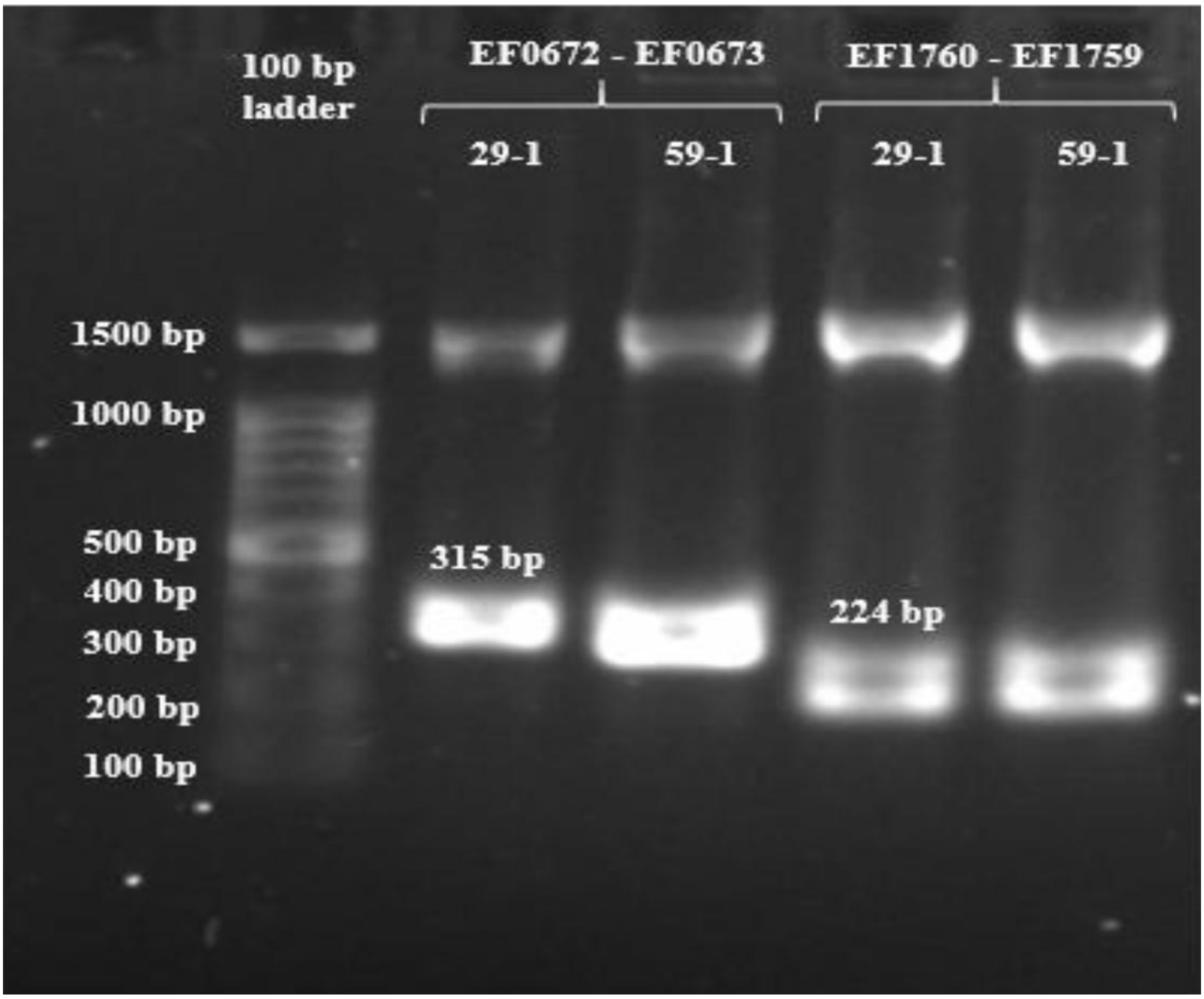

Figure S3: PCR amplification of *E. faecalis* CRISPR1-Cas (EF0672-EF0673) (315 bp) and CRISPR3-Cas (EF1760-EF1759) (224 bp) flanking regions.

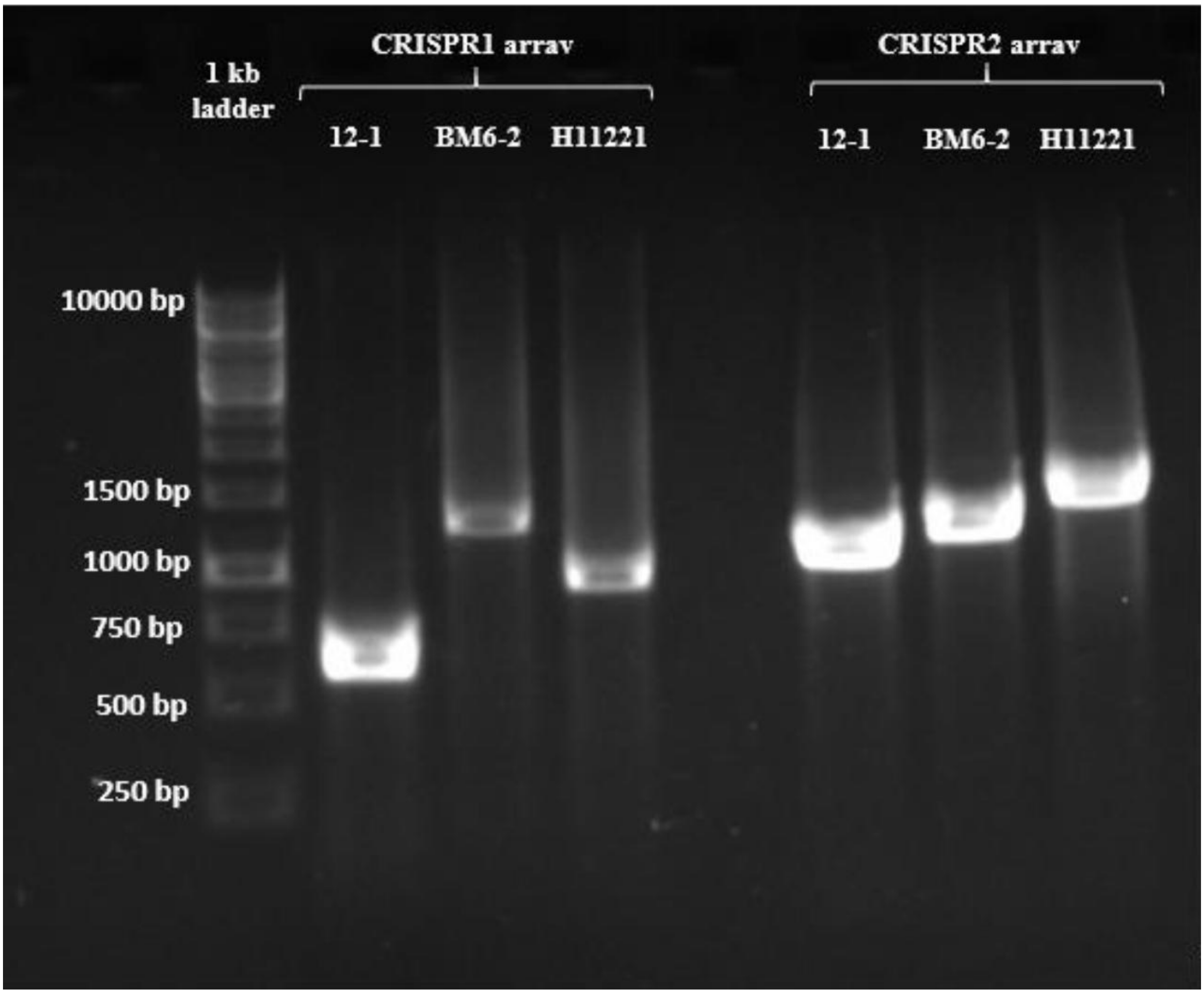

Figure S4: PCR amplification of CRISPR1-Cas and orphan CRISPR2 arrays in *E. faecalis* isolates. The array sizes differ between isolates due to variations in spacer number.

Table S1: PCR cycling protocols for CRISPR1 and CRISPR2 arrays. Primers and conditions for CRISPR2 array were ourced from Palmer & Gilmore [1].

Primers	Primer sequence (5' → 3')	Amplicon size (bp)	Annealing temperature (°C)
CRISPR1 array Forward	GGGTGGTCCAGAACAGATTCC	Variable	50
CRISPR1 array Reverse	TCGTAATCGCATCACTTGCT		
CRISPR2 array Forward	CTGGCTCGCTGTTACAGCT	Variable	58
CRISPR2 array Reverse	GCCAATGTTACAATATCAAACA		

Table S2: Statistical analysis of association between CRISPR-Cas and antimicrobial resistance.

Antibiotics	Susceptibility to antibiotic	CRISPR1-Cas present	CRISPR1-Cas absent	*p*-value[a]
Nitrofurantoin	Resistant	0	0	0.00
	Susceptible	13	13	
Chloramphenicol	Resistant	1	5	0.16
	Susceptible	12	8	
Ciprofloxacin	Resistant	7	4	0.43
	Susceptible	6	9	
Erythromycin	Resistant	7	13	0.02
	Susceptible	6	0	
Penicillin	Resistant	1	0	1.00
	Susceptible	12	13	
Tetracycline	Resistant	3	6	0.41
	Susceptible	10	7	
Linezolid	Resistant	2	2	1.00
	Susceptible	11	11	
Streptomycin	Resistant	1	1	1.00
	Susceptible	12	12	
Gentamicin	Resistant	1	1	1.00
	Susceptible	12	12	
Vancomycin	Resistant	4	1	0.32
	Susceptible	9	12	

[a] $p < 0.05$ indicates significance

References:

1. Palmer KL, Gilmore MS. Multidrug-resistant enterococci lack CRISPR-cas. mBio. 2010;1(4).
